# Supplementary material for: Barriers and Facilitators to Father’s Engagement in a Depression and Alcohol Use Intervention in Kenya: Father, Family, and Community Factors
Source: Int J Environ Res Public Health. 2023 Mar 9;20(6):4830. doi: 10.3390/ijerph20064830 (PMC10048967; doi:10.3390/ijerph20064830)
Supplement: Supplementary file 1 [file ijerph-20-04830-s001.zip › ijerph-2179385-supplementary.pdf]

Focus Group Guide: Fathers

Introduction
Hi, I am ______ from the ________. Thank you for agreeing to take part in this discussion group. I am very excited to be here and learn more about you.

We are interested in hearing about your experiences and understanding how you think others can help other men. We want to understand how to help men who want to change their drinking, whose drinking causes problems, or who feel like they are without purpose and want to feel different. We've gotten this group together today so we can learn more about your experiences and ideas of what might help.

I have some specific questions I'd like to get your thoughts on today. Please feel free to speak openly, everything you tell me will be very important and useful; and there are no right or wrong answers. It is very important that we hear your honest opinions.

You probably prefer that your comments not be repeated to people outside of this group. Please treat others in the group as you want to be treated by not telling anyone about what you hear in this discussion today.

The research team will also treat this discussion confidentially—which means that we will not use your name or anything else that might identify you in our reports. We will also not share what you say with anyone else outside the group, unless you tell us something that makes us fear for your safety.

If there are any questions you don't feel comfortable answering that's okay. Let me know if you need to stop for any reason.

Do you have any questions before we begin?

Part 1: Ice breaker/background

We'll start out by getting to know each other a little bit more.

1. Can you tell me how many children you have (*ask separately for boys and girls*)? In addition – are there any other children you care for who live with you in your home?
2. Are you currently married or living with your partner? For how long have you been living with her/him [current partner]?
3. What is your favorite song right now?

Part 2: General thoughts on Drinking, Mood, and Family Problems
[The goal here is to understand how men see these problems in their selves and others. We also want to understand 1) what they see as the biggest problems, 2)the ways they have tried to make things better, 3) what or who has helped, 4) what or who has gotten in the way, and 5) the reasons for change]

Now I'd like to talk a little bit about your experiences and ideas about men and father's mental health, drinking, and their families.

1. What does the term mental health mean to you?

- Think of someone you now with good mental health, what do they act like?
- Think of someone you know with poor mental health, what do they act like?

2. We all deal with problems. Besides money, what are the biggest problems that get in the way of your goals?
- If a miracle happened, what would life look like? How would you feel?
    - How is that different from how you feel now?
- What things do you do that help you feel better or make you feel worse?

3. ***Drinking:*** People drink for different reasons, tell me why you drink.
Some people want to change their drinking, some people do not.
    - If you want to change your drinking, what are some reasons you want to make this change?
        - Tell me what makes it hard to change.
    - If you do not, tell me why you do not want to change?

    - Tell me about ways you or others have been able to stop or reduce drinking. What works best?
    - Tell me about ways that being a man (cultural beliefs) in your community might make it harder or easier to make changes?

4. **Mood:** Many men tell us that they often feel idle, down, worried or that they are thinking too much. Sometimes this leads them to drink and sometimes this makes them feel like they cannot do anything.
    - (Describe) Tell me about you or others you have seen who have these feelings.
    - Tell me about ways that you or others try to feel better?
        - What has worked
        - What does not work?
        - Are there ways that the ways you are expected to be a man in your community (cultural beliefs) makes it hard to make changes or ask for help?

5. Family Problems: Drinking and feeling low can sometimes lead to problems in the family. What are the biggest problems you see in the family caused by these problems?
    - How do you think these problems hurt or help children in the family (tell me about that)?
    - Tell me about ways you or others have been able to change problems in the family?
    - **How do families help or stop men from making changes?**

Part 3: Help and Interventions

Sometimes when people want to make change or feel different they look for help. We want to ask you questions to understand how to best help men who want to make changes. We want to understand: 1) what types of programs or skills might help, 2) who are the best people to provide the program, 3) where is the best place to hold the program, and 4) the best format for the program.

1. ***Who:*** If you or someone you know are having any of these problems (family, drinking, depression) and you wanted to change them, tell me about who you would turn to for help?
    [Probe: Health care workers? Teachers? Other family members? Neighbors? Local Council Leaders? Religious Leaders? Other men?] ***Why?***
        [OR: what *type* of person (e.g., not harsh, not shaming).
    - Are there certain people you would **never** talk to about these problems? Why?
    - Who do you think are the best people to help with these problems and teach men new ways to change problems?

[Probe: other fathers, other men, role models, counselors, hospital workers, women, young, old, from or outside your community; ***List out who the fathers note***]

- Tell me about what it would be like to have other fathers from the community to talk to versus someone who works at the hospital?

[***Have group rank 1-5 top options***]

2. ***<u>Where</u>***: If there was program to help with these problems, tell me where people should meet to talk about these problems and learn new skills? Why? Tell me where would be bad places to meet? Why? [Probe: school, common community area, office space, hospital, clinic, church/mosque, home, other?]

- Some programs are provided one-on-one (person and program leader), some happen in a group (other men with similar goals), and some happen with family members? Tell me what you think would be best for helping make changes? [Or some combination of these formats]
  - Probes: Why, how or who from family would be involved. Can you give me an example.
- What time of day do you think is best?
- When and how long should the program be?

[***Have group rank 1-5 top strategies i.e., what is most important]***

3. **Engagement**: Sometimes it is difficult to get men to come to programs. Tell us some of your ideas for telling men about the program and encouraging them to come to the program. [Note: we cannot offer money, so probe for more answers if this comes up]

4. **Sustainability**: Sometimes programs stop and start for many reasons, what are your ideas for helping a program last a long time in your community?

[**BREAK**]

You've given some great information so far, thank you. Next, I want to get your ideas about skills and topics that have been shown to help men who want to change drinking and feel better.

Would it be okay for me to tell you some of the activities that have helped men feel better? I will talk about a few different activities then I want us to try and rank what might be the most and least useful. Does that make sense? Great.

1) The first is **<u>Value Activities</u>**: this means choosing something that is important to you and planning activities that fit with that value. This has been shown to reduce drinking and help positive feelings.
2) **Man Talk**: In this activity we talk about what it means to be a man and good father. This helps us add ideas to how we think about being a man and talk about people we look up to and don't look up.
3) **How to say no**: Here we talk about and practice how to say no to friends that want you to drink.
4) **Why I Drink:** Here we help men understand clues to what feelings and situations lead to a push to drink or be alone. Understanding these paths can help us choose new steps to take.
5) **Who to talk to**: Identify who can help you on your journey to make change.

These are activities that have helped other men in this community. Are there other activities you would want to include? [List]

- [Can add the following if not brought up: How to communicate better with family members, talking about finances, knowledge of parenting practices, exercises to reduce thinking too much]

I think we have a great list. Which of these activities sound most interesting or helpful? [**Rank 1-5]**

Closure

Thank you for so much for all of your valuable thoughts. Is there anything else you would like to share before we end today?

Thank you again. What we discussed was not easy but really important, and I really admire willingness to share. It will be very helpful for us in developing programs to make adolescents across the country feel better. [Give incentives]

Key Informant Interview: Policy Maker/Community Leader/Hospital Leadership

Introduction
Hi, I am ______ from ________. Thank you for agreeing to take part in this interview. I am very excited to be here and learn from you.

We are interested in understanding how we can deliver care for men with mental health and drinking problems in Eldoret whose problems hurt their families. Specifically, we want to understand how we can reach men with these problems, help them get help, and deliver a program that we hope will last a long time.

We wanted to gain your perspective given you are an expert, and we are interested in knowing what you think about men's mental health and drinking services as well as your thoughts on incorporating new mental health services in the community and/or MTRH. I have some specific questions I'd like to ask you related to this. Your answers will inform how a treatment for these problems will be delivered.

The research team will also treat this discussion confidentially—which means that we will not use your name or anything else that might identify you in our reports.

Do you have any questions before we begin?

Participant Information

1. First could you please tell me the title of your position and what you do as part of it?
    - How many years have you been in this position?

Part 1: General thoughts on Men's Drinking, Mood, and Family Problems
First, I'd like to talk a little bit about your experiences with men's mental health and drinking.

2. Thinking about men and fathers in your community, what types of mental health and drinking problems do you see?
    - Tell me about some of the challenges these men and families face.
    - Tell me what you think is the hardest thing for men to change or get help for. [Probe: emotion regulation (staying calm), drinking, staying busy, interacting with family]
    - What usually happens with these families, do they ask for help or usually not?

Part 2: Current Mental Health Care Services [Goal: understand how men seek and find help, what services they usually provide, the gaps they see in care and what works well in care]

1. [IF they work in a health system] **How are men identified as feeling very down (depressed) or having drinking problems?** [Probe: Are they given measures? Interviewed (if so how)?]
    - What are the problems with this system?
    - What works well in this system?

2. **Tell me about how men usually find help if they are feeling very idle, drinking too much, or having family problems?** [Probe: Do they find you, do people bring them, outreach efforts? Pathways may be different for the different problems]
    - What works well with this system?
    - What gets in the way of men finding help?

  - [Economic, political] *Policies or lack of services?*
  - [Inner Setting] *What works **well** with them getting services at the hospital? What **does not?*** [Probe: organization, hospital culture, stigma?]
- Tell me about what services you are aware of for these problems?

- Sometimes men ask for help and sometimes they do not. **What do you think stops men from getting help for mental health? What do you think might encourage them to seek help?**

3. Tell me about your opinion of the current treatment options for men with mental health problems?
   - What works well?
   - Tell me how you would change things or add things?

5. Men can sometimes be hard to engage in treatment or help due to work and cultural reasons. For example, some men might think it is not manly to come get mental health care.
   - Tell me about a time you might have seen this in your community.
   - What ideas do you have that might help programs engage men in new services? [Probe: name, who delivers, where, relationship specifics, other creative ideas!]

6. Can you think of an example of a new program in your community/hospital that was successfully delivered over time? Tell me about what worked will to deliver that program.

7. Tell me about certain people you believe would be important to contact that may champion a program for men's mental health (e.g., someone who can advocate for the program)?

Part 4: Intervention Exploration
Thank you so much for all your thoughts and ideas already. Next, I want to tell you a little bit about a treatment for men with problem drinking, low mood, and family problems that has been used and tested in Eldoret. The treatment is called LEAD which stands for Learn, Engage, Act, and Dedicate. It was developed to be used in Eldoret. It is 5 sessions, and patients meet with a counselor 1-on-1. In the past peer-fathers who were nominated by community leaders provided the treatment in an office location.

I would love your ideas on how to best deliver the treatment in the community or hospital in ways that the program can be sustained. In other words, how it can last. This includes: 1) finding and identifying men with these problems, 2) getting them to come to treatment, and 3) delivering treatment.

1. What system might be best to connect with to identify and deliver this type of treatment: community leaders or organizations, the hospital, or both? Why?
   - How would it take place?
   - Where would it take place?
   - What type of partnerships need to be made?
   - What administrative approvals would be required?

   - What are the barriers to delivering in the hospital? What are the positives?
     - In the hospital, can you think of an example of a mental health program.
       - IF YES:
         - How is this program evaluated?
         - How does the public know about the program?

            - How is the program funded?
- What are the barriers to delivering in the community? What are the positives?
    - In the community, can you think of an example of a mental health program.
        - IF YES:
            - How is this program evaluated?
            - How does the public know about the program?
            - How is the program funded?

2. What resources exist to help support this type of program (Probes: policies/legislation, financial and human resources)? What is a barrier to delivering this program?

3. Describe the type of person you think would be good to deliver this kind of treatment? [Probe: qualities, training, gender; in the past we have used peer-fathers]

- Tell me about the good things and the challenges of having someone from the community deliver the treatment versus someone affiliated with a school or the hospital (e.g., peer-provider or professional)?

5. Tell me your thoughts on having men meet one on one with a counselor versus in a group? [Probe: If the topic of family comes up, ask pros and cons of including family members; *If group ask why and the challenges with a group for men with more severe problems*]

6. Lastly, please tell me if you have any thoughts on how to keep a program like this going once it starts?

Closure
Thank you for sharing today. Your replies are very important and will be very useful for our future programming. Is there anything else you would like to share with us today?
Thank you again. (Give incentives)

Please leave your name with us if it is okay for us to contact you again to ask your feedback in delivering the program.

Key Informant Interview: Mental Health Providers

Introduction

Hi, I am ______ from __________. Thank you for agreeing to take part in this discussion group. I am very excited to be here and learn more about you.

We are interested in understanding how we can deliver more outpatient care for men with mental health and drinking problems in Eldoret. Specifically, we want to understand if it is possible to identify (screen) men with depression and drinking problems and engage them in therapy.

We wanted to interview you because of your expertise providing care in this area and working within this system. We are interested in knowing what you think about men's mental health and drinking as well as your thoughts on incorporating new mental health services in the community or MTRH. I have some specific questions I'd like to ask you all today related to this.

The research team will also treat this discussion confidentially—which means that we will not use your name or anything else that might identify you in our reports unless you would like us too.

Do you have any questions before we begin?

Participant Information

- First could you please tell me your job title and briefly what you do in this position?

Part 1: Introduction/Ice breaker

Please tell us how many years you have worked at [____] and the first word that comes to mind when you think about fathers and mental health.

Part 2: General thoughts on Men's Drinking, Mood, and Family Problems

First, I'd like to talk a little bit about your experiences and ideas about mental health and drinking particularly among men and fathers. It would be most helpful to hear about men who have problems drinking but who are not dependent on alcohol.

3. Thinking about the men who come to you, what types of mental health and drinking problems do you think exist?
    - Tell me about some of the challenges these men and families face
    - Tell me what you think is the hardest thing for men to change? [Probe: emotion regulation (staying calm), drinking, staying busy, interacting with family]
    - What usually happens with these men and families, do they ask for help or usually not?

Part 3: Current Mental Health Care Services [Goal: understand how men seek and find help, what services they usually provide, <u>the gaps they see in care and what works well in care</u>]

1. Tell me about how a man might find or come to your services? [Probe: Do they find you, do people bring them, outreach efforts?]

- What works well with this system?
- What is challenging or what would you like to change about how men in need find services?

- How do you think men are identified as needing help outside your care systems? [Probe: For example, sometimes community leaders or family members may identify a problem. Can they help connect men to care? What are the challenges?]

2. **How are men identified as feeling very down (depressed) or having drinking problems?** [Probe: Are they given measures? Interviewed (if so how)?]
- What are the problems with this system?
- What works well in this system?

- Tell me how you would feel about administering short measures/questionnaires to identify men with low mood and/or drinking problems?
    - What would make this difficult?
    - Tell me how you think a brief questionnaire to identify problems could be administered (probes: where, by whom?)? [*Make sure this means using a measure to ID problems*]

3. After a man is identified as needing help, what services are provided or offered? [Probe: strategies used, description of service provided, *are different services offered for drinking and depression?]*
- If a man has both depression and drinking problems, what happens?
    - Tell me what his treatment or connection to services might look like?
- Tell me what you would add or change to this process?

4. How do you feel about the current treatment options for men with mental health problems?
- What is missing or what would you add?
- What works well?
- Tell me how you would change things?

5. Men can sometimes be hard to engage in treatment (hard for them to keep coming) due to work and cultural reasons. For example, some men might think it is not manly to come get mental health care.
- Tell me about a time you have had this challenge.
- Tell me what you do to help engage men specifically in treatment.
- What ideas do you have that might help other programs engage men in new services? [Probe: name, who delivers, where, relationship specifics, other creative ideas!]

Part 4: Intervention Exploration
Thank you so much for all your ideas already. Next, I want to tell you a little bit about a treatment for men with problem drinking, low mood, and family problems. After telling you a bit more about the treatment, I would love your ideas on 1) how to best engage men in such a program, 2) who should deliver treatment, 3) where treatment should be delivered, 4) how to deliver treatment (one-on-one, group), and 5) what you feel like is missing or may need to be changed in the program.

Great. Briefly, the program that has been used for these problems is call LEAD which stands for Learn, Engage, Act, and Dedicate. It is for fathers whose drinking and sad feelings lead to problems in their family. LEAD is 5 sessions, with 1 session a week. It was developed to be used in Eldoret, and was tested here with promising results. It starts with 1 session of motivational interviewing to engage men in treatment, increase their readiness to change, and identify two values that are important to them- one for themselves, like be a good leader, and one for their family, like be a good father. In the next sessions, they use these values to pick new activities to replace and reduce drinking and improve their mood.

Studies have shown that this is an effective way to do both. They also learn what feelings and situations give them the urge to engage in drinking, that way they can learn to choose new behaviors when they notice these events or urges. LEAD includes a few more components: 1) refusal skills (how to say no to people asking you to drink), 2) talking about what it means to be a man and good father to help men grow ideas of what it means to be a man in the family beyond just a provider but also a care-er, 3) identifying people who can help you stay on you positive, healthy path. LEAD was delivered by other fathers in the community who were seen as role models.

1. Hearing this, tell me you initial thoughts?
    - What do you think would work well in this program?
    - What would you take away, add, or change?

2. Describe the type of person you think would be good to deliver this kind of treatment? [Probe: qualities, training, gender]
    - Tell me about the good things and the challenges of having someone from the community deliver LEAD versus someone affiliated with a school/hospital (e.g., lay person vs. professional?

3. If you received training, how would you feel about being a supervisor or provider in this treatment? *Why*?

4. Tell me your thoughts on having men meet one on one with a counselor versus in a group? [Probe: If the topic of family comes up, ask pros and cons of including family members; *If group ask why and the challenges with a group for men with more severe problems*]

5. Where do you think men would feel most comfortable attending a program? Why?

Closure

Thank you for sharing today. Your replies are very important and will be very useful for our future programming. Is there anything else you would like to share with us today?
Thank you again. (Give incentives)
Please leave your name with us if it is okay for us to contact you again to ask your feedback in delivering the program.

Focus Group Guides: Alcohol Use and Mental Health Providers

Note to the Facilitator: *Bring notecards or sticky notes for ranking. Take notes about key ideas throughout. Remember the audio recorder! Bring extra consent form in case any person has not yet complete consent. Bring needed compensation for any travel reimbursement and refreshments.*

Introduction
Hi, We are ______ from the ________. Thank you for agreeing to take part in this discussion group. I am very excited to be here and learn more about you.

We are interested in understanding how we can deliver more outpatient care for men with mental health and drinking problems in Eldoret from many different perspectives. Specifically, we want to understand if it is possible to identify (screen) men with depression and drinking problems and engage them in therapy.

We wanted to hear from all of you because you each have expertise providing care in this area and working within this system in different ways. We want to hear what each of you think and bring together your ideas to think of a way forward for these issues. We are interested in hearing what you each think about men's mental health and drinking as well as your thoughts on how to best incorporate a new mental health program for men's drinking and depression in the community or MTRH.

We/I have some specific questions we'd like to ask you all today related to this. It is very important to be honest and to share your point of view, regardless of whether you agree or disagree with what you hear. There are no right or wrong answers in this discussion. In fact, we hope to hear different opinions and work together to find a way forward because all the opinions matter. Each of you work in different places and do different tasks, each of what you do is important and we want to hear all of your thoughts. We ask everyone is respectful of those around them and give time for all to speak. We think this will lead to a wonderful conversation.

You probably prefer that your comments not be repeated to people outside of this group. Please treat others in the group as you want to be treated by not telling anyone about what you hear in this discussion today. The research team will also treat this discussion confidentially—which means that we will not use your name or anything else that might identify you in our reports unless you would like us too.

If there are any questions you don't feel comfortable answering that's okay. Let me know if you need to stop for any reason.

Do you have any questions before we begin?

Participant Information
- First could you please tell me your job title and briefly what you do in this position?

Part 1: Introduction/Ice breaker
1. Please tell us how many years you have worked at [____], what do you do during your free time when not at work, and what comes to mind when you think about fathers and mental health. I will go first…. [*researcher goes first to set a one word example mentioning something that can stimulate discussion – e.g., preparation, important, difficult. Keep this short!]*

Part 2: General thoughts on Men's Drinking, Mood, and Family Problems
First, I'd like to talk about your experiences and ideas about mental health and drinking particularly among men and fathers. **It would be most helpful to hear about men who have problems drinking, like those getting in fights with their families about their drinking but who are not the most addicted to alcohol so don't need it to wake up in the morning or sleeping in the streets.**

1. Thinking about the men who come to you, what types of mental health and drinking problems do you think exist?
    - Tell me about some of the challenges these men face.
    - Of these challenges that men come with, which ones do you think are the hardest for men to change? Why so.
    - What usually happens with these men and families, do they ask for help or usually not?

Part 3: Current Mental Health Care Services [Goal: understand how men seek and find help, what services they usually provide, <u>the gaps they see in care and what works well in care</u>]

1. Tell me about how a man might find or come to your services (note your services in your answer)? [Probe: Do they find you, do people bring them, outreach efforts?]

- What works well with this method of men coming to your service
- What is challenging or what would you like to change about how men in need find services?
- How do you think men are identified as needing help outside your service? [Probe: For example, sometimes community leaders or family members may identify a problem. Can they help connect men to care? What are the challenges?]

2. **How are men in your service identified as feeling depressed or having drinking problems?** [Probe: Are they given measures? Interviewed (if so how)?]

- What works well in this system?
- What are the problems with this system?
- Tell me how you would feel about administering short measures/questionnaires [4-20 items] to identify men with low mood and/or drinking problems?
    - o What would make this difficult?
    - o Tell me how you think a brief questionnaire to identify problems could be administered (probes: where, by whom?)? [*Make sure this means using a measure to ID problems*]

3. After a man is identified as needing help, what services are provided or offered? [Probe: strategies used, description of service provided, *are different services offered for drinking and depression?]*

- If a man has both depression and drinking problems, what happens?
    - o Tell me what his treatment or connection to services might look like?
- Tell me what you would add or change to this process?

4. How do you feel about the current treatment options for men with mental health problems and alcohol use problems?

- What is missing or what would you add?
- What works well?
- Tell me how you would change things?

5. Sometimes it can be hard to bring men to mental health treatment (get them to come) and get them to keep coming due to work and/or cultural reasons. For example, some men might think it is not manly to come get mental health care.

- Tell me about a time you have had a challenge like this. [Bringing men to care, keeping men]
- Tell me what you do to help bring and keep men specifically in treatment.
- **Tell me about ways you have seen others bring and keep men engaged in mental health care?**

Part 4: Intervention Exploration

Thank you so much for all your ideas already. Next, I want to tell you a little bit about a treatment for men with problem drinking, low mood, and family problems. After telling you a bit more about the treatment, I would love your ideas on 1) how to best engage men in such a program, 2) who should deliver treatment, 3) where treatment should be delivered, 4) how to deliver treatment (one-on-one, group), and 5) what you feel like is missing or may need to be changed in the program.

Great. Briefly, the program that has been tried for these problems is call LEAD which stands for Learn, Engage, Act, and Dedicate. It is for fathers whose drinking and sad feelings lead to problems in their family. LEAD is 5 sessions, with 1 session a week. It was developed to be used in Eldoret, and was tested here with promising results. It starts with 1 session of motivational interviewing to engage men in treatment, increase their readiness to change, and identify two values that are important to them- one for themselves, like be a good leader, and one for their family, like be a good father. In the next sessions, they use these values to pick new activities to replace and reduce drinking and improve their mood. Studies have shown that this is an effective way to do both. They also learn what feelings and situations give them the urge to engage in drinking, that way they can learn to choose new behaviors when they notice these events or urges. LEAD includes a few more components: 1) refusal skills (how to say no to people asking you to drink), 2) talking about what it means to be a man and good father to help men grow ideas of what it means to be a man in the family beyond just a provider but also a care-er, 3) identifying people who can help you stay on your positive, healthy path. LEAD was delivered by other fathers in the community who were seen as role models. They were trained on the treatment and were provided supervision throughout.

1. Hearing this, tell me you initial thoughts? [Can be brief here]
    - What questions do you have about it?
    - What do you think about this program?
    - What would you take away, add, or change?

2. Describe the type of person you think would be good to deliver this kind of treatment? [Probe: qualities, training, gender]
    - Tell me about the good things and the challenges of having someone from the community deliver LEAD versus someone affiliated with a hospital (e.g., lay person vs. professional)?

3. If you received training, how would you feel about being a supervisor or provider in this treatment? *Why*?

4. Tell me your thoughts on having men meet one on one with a counselor versus in a group? [Probe: If the topic of family comes up, ask pros and cons of including family members; *If group ask why and the challenges with a group for men with more severe problems*]

5. Where do you think men would feel most comfortable attending a program? Why?

6. What do you think can ensure men attend all the treatment sessions? Why?

7. After men complete LEAD, which services do you think are think we can connect men to care to that can help them keep changes?

- If nothing exists, what would you create?

Part 5: List and Rank [Use the options they have come up with as well as what is in the lists]. We have talked about many things! Now I want us to spend the last of our time ranking some of our ideas from 1 to 5 regarding such a program. It will be common that we might not agree on the exact position of things but we will do our best to come to consensus.

Screening/Id'ing Men: Presenting to the hospital; asking community leaders for help; other clinics; community centers; religious centers; community lead ID; family member ID; self-referred; other patient's ID….
Setting: Hospital; office space; community center; school; other center?; field; home; ….
Format: One-on-one; group; some family member involvement; no family member involvement
Provider Qualities: male/female; open; warm; good listener; take feedback well; want to learn; available; same community, different community;
Engagement Strategies: *Ask for top idea for engaging men in treatment. If money comes up, think of at least one alternative.*

Closure
Thank you for sharing today. Your replies are very important and will be very useful for our future programming. Is there anything else you would like to share with us today?
Thank you again. (Give incentives)
Please leave your name with us if it is okay for us to contact you again to ask your feedback in delivering the program.

Key Informant Interview: Past Peer-Father Counselors

Introduction

Hi, I am ______ from ___________. Thank you for agreeing to talk again! It has been so long, and I am so happy you agreed to speak with me again about your past counseling experiences.

We now might be able take the program you learned and deliver it to more people and train more counselors. Since you are an expert in the program, we are interested in understanding how we can better deliver the leadership and counseling program to men who are feeling idle and want to reduce their drinking in Eldoret. We wanted to hear about your experiences after the program and any ideas you had for making it better and easier for others. We would also like to invite you to be on a community advisory board that will help make decisions about the program if we are able to get funding to deliver it in the community or hospital.

I have some specific questions I'd like to ask you all today related to this.

If there are any questions you don't feel comfortable answering any questions that's okay. Let me know if you need to stop for any reason.

Do you have any questions before we begin?

Participant Information

2. First could you please tell me the area where you live, your position, and your favorite song?

Part 1: Introduction/Program Changes, Lessons

1. Please tell me about what you remember from the past leadership and counseling program.
- Tell me what you would change [if you recall].
- Tell me what you would keep the same [if you recall].
- Tell me what you think helped men come to the sessions
- Tell me what made it hard to come to the sessions

2. We know a big problem with this program is that it stopped quickly once the project money was over. Tell me any ideas you have that can help keep a program like this going and train counselors besides project money?
- Tell me about ways that counselors might want to participate in addition to their jobs?
- Tell me how many patients you think a counselor could take on at once?

Part 2: Delivery and Program Changes

3. Tell me about where you think a program should be offered so that men feel comfortable going.
   [Probe: hospital, office, community center, home]

5. You conducted this program 1-on-1. Tell me what you liked about this. What did you not like about it.
- Tell me your thoughts on doing such a program in a group? [Probe: pros/cons]
  - What about with other family members? [Probe: pros/cons]
    - What would be the pros and cons of including spouses?
      - What about children?

6. Tell me what you remember about being a counselor.
- What did you like about it?
- What would you change?

7. Tell me about what you think the positives and negatives are about having someone from the community who is also a father deliver the program?
- What do you think was good about this?
- Tell me who you think a man with drinking problems might feel most comfortable talking to: someone who works at a hospital, school, church/mosque, or a peer or someone else? Why?

8. If we are able to get funding to deliver the program again, would you be interested in being involved?

Closure
Thank you for sharing today. Your replies are very important and will be very useful for our future programming. Is there anything else you would like to share with us today?
Thank you again. (Give incentives)

Would it be okay for us to contact you again to ask your feedback in delivering the program.

Key Informant Interview: Past Patients

Introduction
Hi, I am ______ from the _________. Thank you for agreeing to take part in this discussion. I am happy you agreed to speak with me about your past counseling experiences.

We are interested in understanding how we can better deliver more leadership and counseling programs to men who are feeling idle and want to reduce their drinking in Eldoret. We wanted to interview you because of you took part in a program that tried to do this in 2018. We wanted to hear about your experiences after the program and any ideas you had for making it better and easier to attend for others.

I have some specific questions I'd like to ask you all today related to this.

The research team will also treat this discussion confidentially—which means that we will not use any your name or anything else that might identify you in our reports. We will also not share what you say with anyone else outside the group, unless you tell us something that makes us fear for your safety.

If there are any questions you don't feel comfortable answering that's okay. Let me know if you need to stop for any reason.

Do you have any questions before we begin?

Participant Information
First could you please tell me how many children you have and the ages of the oldest and youngest?

Part 1: Introduction/Program Changes, Lessons

1. Please tell me about what you remember from the past leadership and counseling program
   - Tell me what you would change
   - Tell me what you would keep the same

   - Tell me what helped you come to the sessions
   - Tell me what made it hard to come to the sessions

2. After these programs sometimes people feel better for a while and stay that way or some people feel a bit better then feel worse again. Tell me now that time has passed have there been any changes that have remained?
   - Tell me about what made it hardest to stay on the path
   - Tell me about what might be helpful to stay on the path
   - Tell me any lessons that you might remember from the program (it is okay if there are none)
     - Why do you think those lessons stuck with you?

Part 2: Identification and Delivery

3. Tell me how you think men should find help if they need it? Should someone approach them about programs or how can they make it easy and comfortable for men to find programs?
   - Would you prefer a community member, family member to inform you of a program?

4. Tell me about **where** you think a program should be offered so that men feel comfortable going?
[Probe: hospital, office, community center, home]

5. You had your program one on one with a counselor. Tell me what you liked about this. What did you not like about this.

- Tell me your thoughts on doing such a program in a group? [Probe: pros/cons]
  - What about with other family members? [Probe: pros/cons]

6. Tell me what you remember about your counselor.

- What did you like about your counselor?
- What would you change?
- Do you prefer having someone you know or don't know counsel you?
- Do you prefer someone from the community or the hospital?

Program Changes

7. Do you wish the program was shorter or longer?

8. Tell me what you think about the following: **[**Probe for pros/cons]

- Entering a group with other men who completed the program after finishing
- Becoming a program ambassador and inviting other men to the program
- Learning how to teach some of the program to other fathers

Closure

Thank you for sharing today. Your replies are very important and will be very useful for our future programming. Is there anything else you would like to share with us today?
Thank you again. (Give incentives) [**Provide list of referrals if needed]**

Would it be okay for us to contact you again to ask your feedback in delivering the program.

Focus Group Guide: Community Leaders

Introduction

Hi, I am ______ from the Moi Teaching and Referral Hospital. Thank you for agreeing to take part in this discussion group. I am very excited to be here and learn more about you.

We are interested in understanding how we can deliver care for men with mental health and drinking problems in Eldoret whose problems then hurt their families. Specifically, we want to understand how we can reach men with these problems and help them get help.

We have brought you here for this expert panel because we are interested in knowing what each of you think about men's mental health and drinking as well as your thoughts on incorporating new mental health services in the community and/or MTRH.

I have some specific questions I'd like to ask you all today related to this. It is very important to be honest and to share your point of view, regardless of whether you agree or disagree with what you hear. There are no right or wrong answers in this discussion.

You probably prefer that your comments not be repeated to people outside of this group. Please treat others in the group as you want to be treated by not telling anyone about what you hear in this discussion today.

The research team will also treat this discussion confidentially—which means that we will not use any your name or anything else that might identify you in our reports. We will also not share what you say with anyone else outside the group, unless you tell us something that makes us fear for your safety.

If there are any questions you don't feel comfortable answering that's okay. Let me know if you need to stop for any reason.

Do you have any questions before we begin?

Participant Information

First could you please tell us your name, where you reside, and your position in the community?

Part 1: Introduction/Ice breaker

1. Please tell us how many years you have been in this position, how you would describe what you do in your community, and the first word that comes to mind when you think about fathers and mental health . I will go first…. [*researcher goes first to set a one word example mentioning something that can stimulate discussion – e.g., preparation, important, difficult. Keep this short!]*

Part 2: General thoughts on Men's Drinking, Mood, and Family Problems

First, I'd like to talk a little bit about your experiences and ideas about mental health and drinking particularly among men and fathers.

4. Thinking about fathers in your community, what types of mental health and drinking problems do you think exist?
    - Tell me about some of the challenges these men and families face
    - Tell me what you think is the hardest thing for men to change? [Probe: emotion regulation (staying calm), drinking, staying busy, interacting with family]

- What usually happens with these families, do they ask for help or usually not?

Part 3: Current Mental Health Care Services [Goal: understand how men seek and find help, what services they usually provide, the gaps they see in care and what works well in care]

1. **How are men identified as feeling very down (depressed) or having drinking problems?**
   - What are the problems with this system?
   - What works well in this system?

   - Tell me how you would feel about training in using questionnaires to identify men with low mood and/or drinking problems?
     - What would make this difficult?
     - What might be beneficial about this? Why? [Probe: What supports would be needed to help support a process]

2. Tell me about how men might find help if they are feeling very idle, drinking too much, or having family problems? [Probe: Do they find you, do people bring them, outreach efforts? Pathways may be different for the different problems]

- What works well with this system?
- What gets in the way of men finding help?
  - [Economic, political] *Policies or lack of services?*
  - Cultural norms and beliefs?
  - [Inner Setting] *What is works well with them getting services? What does not?* [Probe: organization, hospital culture, stigma?]
- What do you think could encourage them to seek help?

- Tell me what help usually looks like for these men. [Probe: hospital, therapy, talking to religious leader, talking to leader]

3. How do you feel about the current treatment options for men with mental health problems?
   - What is missing or what would you add?
   - What works well?
   - Tell me how you would change things?

5. Men can sometimes be hard to engage in treatment or help due to work and cultural reasons. For example, some men might think it is not manly to come get mental health care.
   - Tell me about a time you might have seen this in your community.
   - Tell me what you do to help engage men in help that leads to change.
   - What ideas do you have that might help other programs engage men in new services? [Probe: name, who delivers, where, relationship specifics, other creative ideas!]

Part 4: Intervention Exploration

Thank you so much for all your ideas already. Next, I want to tell you a little bit about a treatment for men with problem drinking, low mood, and family problems. After telling you a bit more about the treatment, I would love your ideas on 1) how to best engage men in such a program, 2) who should deliver treatment, 3) where treatment should be delivered, 4) how to deliver treatment (one-on-one, group), and 5) what you feel like is missing for may need to changed in the program.

Does that make sense?

Great. Briefly, the program that has been used for these problems is call LEAD which stands for Learn, Engage, Act, and Dedicate. It was developed to be used in Eldoret and was tested here. It is for fathers whose drinking and sad feelings lead to problems in their family. LEAD is 5 sessions, with 1 session a week. It starts with 1 session to engage men in treatment, increase their readiness to change, and identify two values that are important to them- one for themselves, like be a good leader, and one for their family, like be a good father. In the next sessions, they use these values to pick new activities to replace and reduce drinking and improve their mood. Studies have shown that this is an effective way to do both. They also learn what feelings and situations give them the urge to engage in drinking, that way they can learn to choose new behaviors when they notice these events or urges. LEAD includes a few more components: 1) refusal skills (how to say no to people asking you to drink), 2) talking about what it means to be a man and good father to help men grow ideas of what it means to be a man in the family beyond just a provider but also a care-er, 3) identifying people who can help you stay on you positive, healthy path. LEAD was delivered by other fathers in the community who were seen as role models.

1. Hearing this, tell me you initial thoughts?
- What do you think would work well?
- What would you take away, add, or change?

We are also trying to understand how to best deliver the treatment so that men will be most likely to come. We want to understand whether this is through the community, the hospital, or both.

2. Tell us what approach might be best: community, hospital, both? Why?
- How would it take place?
- Where would it take place?
- What type of partnerships need to be made?
- How do we protect confidentiality?
- What are the barriers to delivering in the hospital? What are the positives?
- What are the barriers to delivering in the community? What are the positives?

3. What resources exist to help support this type of program?

4. Describe the type of person you think would be good to deliver this kind of treatment? [Probe: qualities, training, gender; in the past we have used peer-fathers]
- Tell me about the good things and the challenges of having someone from the community deliver the treatment versus someone affiliated with a school or the hospital?

5. Tell me your thoughts on having men meet one on one with a counselor versus in a group? [Probe: If the topic of family comes up, ask pros and cons of including family members; *If group ask why and the challenges with a group for men with more severe problems*]

Part 5: List and Rank [Use the options they have come up with as well as what is in the lists]. We have talked about many things! Now I want us to spend the last of our time ranking some of our ideas from 1 on regarding such a program.

Screening/Identifying Men: Presenting to the hospital; asking community leaders for help; other clinics; community centers; religious centers; community lead ID; family member ID; self-referred; other patient's ID….
Setting: Hospital; office space; community center; school; other center?; field; home; ….
Format: One-on-one; group; some family member involvement; no family member involvement
Provider Qualities: male/female; open; warm; good listener; take feedback well; want to learn; available; same community, different community;
Engagement Strategies: *Ask for top idea for engaging men in treatment. If money comes up, think of at least one alternative.*

Closure
Thank you for sharing today. Your replies are very important and will be very useful for our future programming. Is there anything else you would like to share with us today?
Thank you again. (Give incentives)
Please leave your name with us if it is okay for us to contact you again to ask your feedback in delivering the program.
